# Supplementary material for: DNMT3B rs2424913 as a Risk Factor for Congenital Heart Defects in Down Syndrome
Source: Genes (Basel). 2023 Feb 24;14(3):576. doi: 10.3390/genes14030576 (PMC10048502; doi:10.3390/genes14030576)
Supplement: Supplementary file 1 [file genes-14-00576-s001.zip › genes-2199454-Supplementary material.pdf]

Table S1. Supplementary material 1. Primers and PCR-RFLP conditions for the genotyping of *DNMT, MTHFR* and *MTRR* genes polymorphisms with their mQTL timepoint

Gene Polymorphisms (dbSNP rs) Position	Forward Primer<br>Reverse Primer	PCR conditions	PCR Product (bp)	Restriction Endonuclease Enzyme	Reference	mQTL* timepoint
**DNMT1<br>+32204 A/G<br>rs2228611<br>exon<br>chromosome 19**	5’-GTACTGTAAGCACGGTCACCTG-3’<br>5’-TATGTTGTCCAGGCTCGTCTC-3’	94 °C (5 min)<br>35x: 94 °C (30s), 55 °C (30s), 72 °C (30s)<br>72 °C (5 min)	261	BcoDI	[50]	Birth<br>Childhood<br>Adolescence<br>Middle Age<br>Pregnancy
**DNMT3A<br>-448 A/G<br>rs1550117<br>promoter<br>chromosome 2**	5’-ACACACCGCCCTCACCCCTT-3’<br>5’-TCCAGCAATCCCTGCCCACA-3’	94 °C (5 min)<br>35x: 94 °C (30s), 55 °C (45s), 72 °C (30s)<br>72 °C (5 min)	358	HpyCH4III	[51]	-
**DNMT3B<br>-579 G/T<br>rs1569686<br>promoter<br>chromosome 20**	5’-GAGGTCTCATTATGCCTAGG-3’<br>5’-GGGAGCTCACCTTCTAGAAA-3’	94 °C (5 min)<br>35x: 94 °C (30s), 49 °C (30s), 72 °C (30s)<br>72 °C (5 min)	225	PvuII-HF	[52]	Birth<br>Childhood<br>Adolescence<br>Middle Age<br>Pregnancy
**DNMT3B<br>-149 C/T<br>rs2424913<br>promoter<br>chromosome 20**	5'-TTGTCCTGAAGCTGGCTACC-3'<br>5'-ACCAGGAGAGAAGCCAACAG-3'	94 °C (5 min)<br>35x: 94 °C (30s), 54 °C (30s), 72 °C (30s)<br>72 °C (5 min)	431	AvrII	[53]	Birth<br>Childhood<br>Adolescence<br>Middle Age<br>Pregnancy
**MTHFR<br>677C>T<br>rs1801133<br>exon<br>chromosome 1**	5’TGAAGGAGAAGGTGTCTGCGGGA3’<br>5’AGGACGGTGCGGTGAGAGTG3’	94 °C (2 min)<br>40x: 94 °C (30s), 62 °C (30s), 72 °C (30s)<br>72 °C (7 min)	198	Hinfl	[54]	Childhood<br>Adolescence
**MTHFR<br>1298A>C<br>rs1801131<br>exon<br>chromosome 1**	5’CTTTGGGGAGCTGAAGGACTACTA3’<br>5’CACTTTGTGACCATTCCGGTTTG3’	92 °C (2 min)<br>40x: 92 °C (60s), 56 °C (60s), 72 °C (30s)<br>72 °C (7 min)	163	Mbo II	[54]	Birth<br>Childhood<br>Adolescence<br>Middle Age<br>Pregnancy
**MTRR<br>66A>G<br>rs1801394<br>exon<br>chromosome 5**	5’CAGGCAAAGGCCATCGCAGAAGACAT3’<br>5’CACTTCCCAACCAAAATTCTTCAACG3’	92 °C (2 min)<br>35x: 92 °C (60s), 56 °C (60s), 72 °C (90s)<br>72 °C (7 min)	150	Afl III	[55]	Birth<br>Childhood<br>Adolescence<br>Middle Age<br>Pregnancy

DNMT – DNA methyltransferase; PCR – polymerase chain reaction; RFLP – restriction fragment length polymorphism; mQTL – methylation quantitative trait loci
*derived from mQTLdb
